# Optimism in Active Learning

**DOI:** 10.1155/2015/973696

**Published:** 2015-11-23

**Authors:** Timothé Collet, Olivier Pietquin

**Affiliations:** ^1^CentraleSupélec, MaLIS Research Group, 57070 Metz, France; ^2^GeorgiaTech-CNRS UMI 2958, 57070 Metz, France; ^3^Université de Lille-CRIStAL UMR 9189, SequeL Team, 59650 Villeneuve d'Ascq, France; ^4^Institut Universitaire de France (IUF), 75005 Paris, France

## Abstract

Active learning is the problem of interactively constructing the training set used in classification in order to reduce its size. It would ideally successively add the instance-label pair that decreases the classification error most. However, the effect of the addition of a pair is not known in advance. It can still be estimated with the pairs already in the training set. The online minimization of the classification error involves a tradeoff between exploration and exploitation. This is a common problem in machine learning for which multiarmed bandit, using the approach of Optimism int the Face of Uncertainty, has proven very efficient these last years. This paper introduces three algorithms for the active learning problem in classification using Optimism in the Face of Uncertainty. Experiments lead on built-in problems and real world datasets demonstrate that they compare positively to state-of-the-art methods.

## 1. Introduction

Traditional classification is a supervised learning framework in which the goal is to find the best mapping between an instance space and a label set. It is based only on the knowledge of a set of instances and their corresponding labels called the training set. To obtain it, an expert or oracle is required to manually label each of the examples, which is expensive. Indeed, this task is time consuming and may as well involve any other kind of resources. The aim of active learning [1] is to reduce the number of requests to the expert without losing performances, which is equivalent to maximizing the performance with a certain number of labeled instances. This can be done by dynamically constructing the training set. Each new instance presented to the expert is thus carefully chosen to generate the best gain in performance. The selection is guided by all the previous received labels. This is a sequential decision process [2].

However, the gain in performance due to a particular instance is not known in advance. This is for two reasons: first, the label given by the expert is not known before querying, and second, the true mapping is unknown. However, those values can be estimated also more and more precisely as the training set grows, because it is the goal of classification to get a good estimate of those values. Still, a low confidence must be put on the first estimations while later estimations may be more trusted. An instance may thus be presented to the expert because it is believed to increase the performances of the classifier, resulting in a short term gain. Or, because it will improve the estimations and help to select better instances in the future, resulting in a long term gain.

This is a very common problem in literature known as exploration versus exploitation dilemma. It has been successfully addressed under the multiarmed bandit problem, as introduced in [3] and surveyed in [4]. In this problem, a set of arms (choices) is considered, where each provides a stochastic reward when pulled (selected). The distribution of rewards for an arm is initially unknown. The goal is to define a strategy to successively pull arms, which maximizes the expected reward under a finite budget of pulls. Several methods have been introduced to solve this dilemma. One of them is the Upper Confidence Bound algorithm, introduced in [5]. It uses the approach of Optimism in the Face of Uncertainty, which selects the arm for which the unknown expected reward is possibly the highest. One notable advantage of those algorithms is that they come with finite-sample analysis and theoretical bounds.

The idea is thus to use Optimism in the Face of Uncertainty for the Active Learning problem in classification. To use this approach, the problem is cast under the multiarmed bandit setting. However, this one deals with a finite number of arms, whereas in classification the instance space may be continuous. In order to adapt it to classification, the instance space is partitioned into several clusters. The goal is thus to find the best mapping between the clusters and the label set, under a finite budget of queries to the expert.

At first, we study the case of independent clusters, where the label given to each cluster only depends on the samples taken in it. We show two algorithms capable of the online allocation of samples among clusters. In this context, we need at least one (or even two) sample in each cluster in order to start favoring one for selection. Thus, the number of clusters must not be too high. This implies using a coarse partition which may limit the maximum performance. The choice of this partition is thus a key issue which has no obvious solution.

Allowing the prediction of each cluster to depend on the samples received in others enables us to use a more refined partition. This makes the choice of the partition less critical. We thus study the case of information sharing clusters. The adaptation of the first case to this one goes through the use of a set of coarse partitions combined by using a Committee of Experts approach. We introduce an algorithm that allocates samples in this context. Doing so, the number of clusters is not limited anymore, and increasing it allows us to apply our algorithms on a continuous instance space. Another algorithm is introduced as an extension of the first one using a kernel.

We start by an overview of the existing methods in active learning in Section 2. Then, in Sections 3–5, we describe the algorithms. We handle the cases of independent cluster and information sharing clusters. For each one of these problem we define a new loss function that has to be minimized. We also define the confidence interval used by our optimistic algorithms. In Section 6, we evaluate the performance of the algorithms in both built-in problems and real world datasets.

## 2. Related Work 

Many algorithms already exist for active learning. A survey of those methods can be found in [6]. Among them, uncertainty sampling [7] uses a probabilistic classifier (it does not truly output a probability but a score on the label) and samples where the label to give is least certain. In binary classification with labels 0 or 1, this is where the score is closest to 0.5. Query by committee [8, 9] methods consider the version space or hypotheses space as the set of all consistent classifiers (nonnoisy classification) and try to reduce it as fast as possible by sampling the most discriminating instance. It finishes when only one classifier is left in the set. Extensions exist for the noisy case, either by requiring more samples before eliminating a hypothesis [10] or by associating a metric to the version space and trying to reduce it [11, 12]. Other algorithms exist that use a measure of confidence for the labels currently given, such as entropy [13] or variance [14]. Finally, the expected error reduction [15–18] algorithms come from the fact that the measure of performance is mostly the risk and that it makes more sense to minimize it directly rather than some other indirect criteria. Our work belongs to this last category. Using an optimistic approach enables us to minimize directly the true risk instead of the expected belief about it.

Other methods also use Optimism in the Face of Uncertainty for active learning. In [19], the method is more related to query by committee since it tries to select the best hypothesis from a set. It thus considers each hypothesis as an arm of a multiarmed bandit and plays them in an optimistic way. In [20], the authors study the problem of estimating uniformly well the mean values of several distributions under a finite budget. This is equivalent to the problem of active learning for regression with an independent discrete instance space. Although this algorithm may still be used on a classification problem, it is not designed for that purpose. Indeed, a good estimate of the mean values leads to a good prediction of the label. However, from the active learning point of view, it will spend effort to be precise on the estimation of the mean value even if this precision is of no use for the decision of the label. Efforts could have been spent to be more certain about the label to give. The importance of having an algorithm specifically adapted to classification is evaluated in Section 6.

## 3. Materials and Methods

The classical multiarmed bandit setting deals with a finite number of arms. This is not appropriate for the general classification problem in which the instance space may be continuous. In order to adapt this theory to active learning, we must first study the case of a discrete instance space, which may come from a discretized continuous space or originally discrete data. At first, we study the case of independent clusters, where no knowledge is shared between neighbors. After that, we will improve the selection strategy by letting neighbor clusters to share information. At the end, by defining clusters that contain only one instance from the pool each, with a good generalization behavior, we are able to apply this theory to continuous data. We may even define externally the relations between instances and use a kernel.

Let us define the following notations. We consider the instance space *X* and the label set *Y*. In binary classification, the label set is composed of two elements, in this work *Y* = {0,1}. The oracle is represented by an unknown but fixed distribution *P*(*y* ∈ *Y*∣*x* ∈ *X*). The scenario considered in this work is pool-based sampling [7]. It assumes that there is a large pool of unlabeled instances available from which the selection strategy is able to pick. At each time step *t*, an active learning algorithm selects an instance *x*_*t*_ ∈ *X* from the pool, receives a label *y*_*t*_ ∈ *Y* drawn from the underlying distribution, and add the pair to the training set. This is repeated up to time *n*. The aim is to define a selection strategy that generates the best performance of the classifier at time *n*. The performance is measured with the risk, which is the mean error that would achieve the classifier by predicting labels.

## 4. Independent Clusters

### 4.1. Partition of the Instance Space

In this section, we focus on the problem of defining a selection strategy with a discrete instance space. Either the space is already discrete or a continuous space is partitioned into several clusters. The following formulation assumes the latter case; otherwise, the same formulation applies for the discrete case if* clusters* are replaced with* instances*. The instance space is thus divided into *K* clusters. The problem is now to choose in which cluster to sample.

Let us define the partition

(1) $$\begin{array}{c} N=\left\{\;X_{1},\ldots ,X_{K}\;\right\} \end{array}$$

with the following properties:

(i) ∀*i* ∈ ⟦1, *K*⟧ : *X*_*i*_ ≠ *∅*, no cluster is empty,
(ii) ∪_*i*=1_^*K*^ *X*_*i*_ = *X*, the clusters cover the whole instance space,
(iii) ∀(*i*, *j*) ∈ ⟦1, *K*⟧^2^ : *i* ≠ *j*⇒*X*_*i*_∩*X*_*j*_ = *∅*, no clusters overlap.

It is important to note that the partition does not change during the progress of the algorithm.

Having discretized the instance space, we can now formalize the problem under a K-armed bandit setting. Each cluster *X*_*k*_ ∈ *N* is an arm characterized by a Bernoulli distribution *ν*_*k*_ with mean value *μ*_*k*_. Indeed, samples taken in a given cluster can only have a value of 0 or 1. At each round, or time step, *t* ≥ 1, an allocation strategy selects an arm *k*_*t*_ ∈ ⟦1, *K*⟧, which corresponds to picking an instance randomly in the cluster *X*_*k*_*t*__ and receives a sample *y*_*k*,*t*_ ~ *ν*_*k*_, independently of the past samples. Let (*w*_*k*_)_*k*∈⟦1, *K*⟧_ denote the weight of each cluster, with ∑_*k*=1_^*K*^ *w*_*k*_ = 1. For example, in a semisupervised context using pool-based sampling, each weight is proportional to the number of unlabeled data points in each cluster, while, in membership query synthesis, the weights are the sizes or areas of clusters.

Let us define the following notations:

(2) $$\begin{array}{c} T_{k,t}=\overset{t}{\underset{s=1}{\sum }}1_{k_{s}=k} \end{array}$$

is the number of times arm *k* has been pulled up to time *t* and

(3) $$\begin{array}{c} {\hat{\mu }}_{k,t}=\frac{1}{T_{k,t}}\overset{T_{k,s}}{\underset{s=1}{\sum }}y_{k,s} \end{array}$$

is the empirical estimate of the mean *μ*_*k*_ at time *t*.

Under this partition, the mapping of the instance space to the label set is limited to the mapping of clusters to the label set. We thus define the classifier that creates this mapping according to the samples received up to time *t*. In this section, the clusters are assumed to be independent. This means that the label given to a cluster can only depend on samples in this cluster. We use the naive Bayes classifier that gives the label

(4) $$\begin{array}{c} f\left(\;x\in X_{k}\;\right)=l_{k,t}=\left[\;{\hat{\mu }}_{k,t}\;\right] \end{array}$$

to cluster *k*, where [·] is the round operator.

### 4.2. Full Knowledge Criteria

The goal is to build an optimist algorithm for the active learning problem. A common methodology in the Optimism in the Face of Uncertainty paradigm is to characterize first the optimal solution. We thus place ourselves in the Full Knowledge setting. In this setting, we let the allocation strategy depend on the true value of *μ*_*k*_ for each cluster, and this defines the optimal allocation of the budget *n*. An optimist algorithm will then estimate those values and allocate samples as close as possible to the optimal allocation. Note that the true values of *μ*_*k*_ cannot be used by the classifier directly but only by the allocation strategy.

In the following sections, we show two full knowledge criteria: data-dependent and data-independent. In the data-independent case, the optimal allocation does not depend on the samples received so far. It can be related to one-shot active learning, as defined in [18], in which the allocation of the budget is decided before sampling any instances. In the data-dependent case, the label given by the classifier at time *t* is also considered. This is related to fully sequential active learning, as defined in [18], where the allocation of the budget is updated after each sample. Note that in both cases, the optimist algorithms built upon those criteria are fully sequential.

#### 4.2.1. Data-Independent Criterion

In this section, we characterize the optimal allocation of the budget *n* depending only on the values of *μ*_*k*_ for each cluster. We want an allocation of the budget that minimizes the true risk of the classifier at time *n*. Here, the risk is based on the binary loss:

(5) $$\begin{array}{c} L_{0/1}\left(\;y,f\left(\;x\;\right)\;\right)=\left\{\begin{array}{cc} 1, & \text{if }f\left(x\right)\neq y,\\ 0, & \text{otherwise.} \end{array}\right. \end{array}$$

Note that this loss is usually hard to use because of its nonconvex nature.

Using the partition *N*, the true risk of the classifier is the sum of the true risks in each cluster

(6) $$\begin{array}{c} R\left(\;f\;\right)=\overset{K}{\underset{k=1}{\sum }}w_{k}R_{k}\left(\;f\;\right)=\overset{K}{\underset{k=1}{\sum }}w_{k}R_{k}\left(\;l_{k,n}\;\right) \end{array}$$

with

(7) $$\begin{array}{c} R_{k}\left(\;l_{k,n}\;\right)=\left\{\begin{array}{cc} 1-\mu_{k}, & \text{if }l_{k,n}=1,\\ \mu_{k}, & \text{if }l_{k,n}=0. \end{array}\right. \end{array}$$

The risk is the mean number of misclassified instances resulting from a particular prediction of labels.

The optimal label the algorithm should assign to arm *k* is [*μ*_*k*_]. This incurs a regret in the true risk $R_{k}([{\hat{\mu }}_{k,n}])-R_{k}([\mu_{k}])$. In order to define an allocation of the samples according to the [*μ*_*k*_] values regardless of their estimates, the regret is expected over all the samples. This gives us the following definition of the loss for classification per cluster, as the expected regret of the true risk in each cluster,

(8) $$\begin{array}{c} L_{k,n}\left(\;\mu_{k},T_{k,n}\;\right)=E\left[\;R_{k}\left(\;\left[\;{\hat{\mu }}_{k,n}\;\right]\;\right)-R_{k}\left(\;\left[\;\mu_{k}\;\right]\;\right)\;\right], \end{array}$$

where the expectation is taken over the samples:

(9) Lk,nμk,Tk,n=E1−μk1μ^k,n=1+μk1μ^k,n=0−1−μk1μk=1−μk1μk=0=2μk−0.5·Pμ^k,n≠μk.

The value to be minimized by our allocation of the budget is then the global loss. It is the sum of losses in each cluster:

(10) $$\begin{array}{c} L_{n}\left(\;{\left(\;\mu_{k}\;\right)}_{k\in ⟦1,K⟧},{\left(\;T_{k,n}\;\right)}_{k\in ⟦1,K⟧}\;\right)=\overset{K}{\underset{k=1}{\sum }}w_{k}L_{k,n}\left(\;\mu_{k},T_{k,n}\;\right). \end{array}$$

The objective is now to define an allocation of the budget that minimizes this loss. However, in order to inverse the loss to retrieve the allocation, as well as to derive the online allocation strategy, the losses in each cluster have to be strictly decreasing with *T*_*k*,*t*_ and convex. This is not the case with these losses. In order to get a more convenient shape, we bound those losses by pseudolosses. The algorithms we build aim to minimize this pseudoloss instead of the loss defined previously. The idea is thus to bound the probability $\mathbb{P}([{\hat{\mu }}_{k,t}]\neq [\mu_{k}])$. We use the fact that the estimated mean in one subset follows a binomial distribution (labels are either 0 or 1). The bounds obtained this way are very tight and equal at a infinitely countable number of points.

Let *ℐ*_1−*p*_(*n* − ⌊*k*⌋, ⌊*k*⌋ + 1) be the cumulative distribution function of a binomial distribution of parameters *n*, *p*. Then,

(11) Pμ^k,n≠μk=1μk=0Pμ^k,n≥0.5+1μk=1Pμ^k,n<0.5=1μk=01−I1−μkTk,n−Tk,n2,Tk,n2+1+1μk=1I1−μkTk,n−Tk,n2,Tk,n2+1.

Note that the probability given above is a step function of *T*_*k*,*n*_/2 and thus is not a strictly decreasing function of *T*_*k*,*n*_. That is not convenient as we require this condition in the later. That is why we bound this probability by bounding the truncated value ⌊*T*_*k*,*n*_/2⌋. Then,

(12) Pμ^k,n≠μk≤1μk=01−I1−μkTk,n2+1,Tk,n2+1μk=1I1−μkTk,n2,Tk,n2+1.

**Figure 1:**
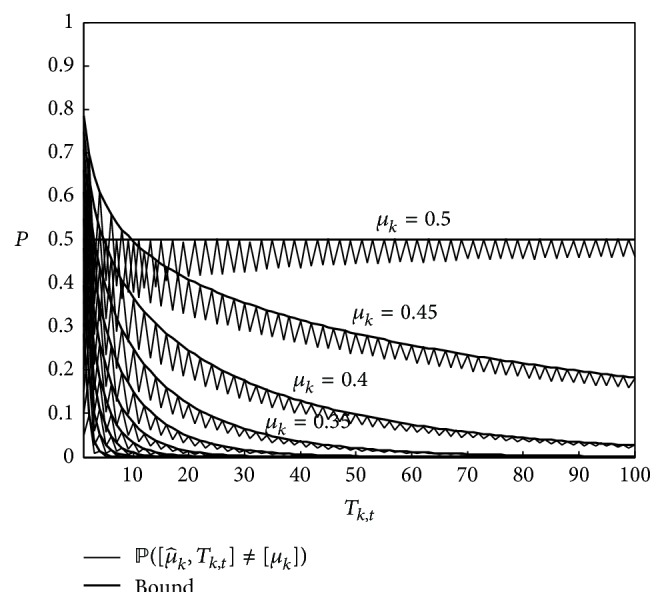
$\mathbb{P}([{\hat{\mu }}_{k,t}]\neq [\mu_{k}])$ and its bound defined in (12).

Figure 1 displays this probability and the corresponding bound function of *T*_*k*,*t*_ for different values of *μ*_*k*_. We can see that the bound is extremely tight, and its only role is to make it strictly decreasing with *T*_*k*,*t*_ and convex. It still retains as much as possible the shape of the probability.

We therefore define the following pseudoloss:

(13) $$\begin{array}{c} {\tilde{L}}_{n}\left(\;{\left(\;\mu_{k}\;\right)}_{k\in ⟦1,K⟧},{\left(\;T_{k,n}\;\right)}_{k\in ⟦1,K⟧}\;\right)=\overset{K}{\underset{k=1}{\sum }}{\tilde{L}}_{k,n}\left(\;\mu_{k},T_{k,n}\;\right) \end{array}$$

with

(14) L~k,nμk,Tk,n=2wkμk−0.5·1μk=01−I1−μkTk,n2+1,Tk,n2+1μk=1I1−μkTk,n2,Tk,n2+1

being the pseudoloss in each cluster.

Due to the convex nature of ${\tilde{L}}_{k,n}$, $\partial {\tilde{L}}_{k,n}/\partial T_{k,n}$ is a strictly increasing function of *T*_*k*,*n*_. Thus, it admits an inverse $({\partial {\tilde{L}}_{k,n}/\partial T_{k,n})}^{-1}$.

Let *T*_*k*,*n*_^*∗*^ be the optimal number of samples to take in each subset in order to minimize ${\tilde{L}}_{n}$ under the constraint that ∑_*k*=1_^*K*^ *T*_*k*,*n*_^*∗*^ = *n*:

(15) $$\begin{array}{c} T_{k,n}^{*}={\left(\;\frac{\partial {\tilde{L}}_{k,n}}{\partial T_{k,n}}\;\right)}^{-1}\left(\;\mu_{k}c^{*}\;\right) \end{array}$$

with *c* ^*∗*^ such that $\sum_{k=1}^{K}{(\partial {\tilde{L}}_{k,n}/\partial T_{k,n})}^{-1}(\mu_{k},c^{*})=n$.

This defines the theoretical optimal allocation of the budget. Since we do not know the closed form for $({\partial {\tilde{L}}_{k,n}/\partial T_{k,n})}^{-1}$ and since an optimist algorithm needs an online allocation criterion, we now show the online allocation criterion *C*_*k*,*t*_,

(16) $$\begin{array}{c} C_{k,t}^{i}\left(\;\mu_{k},T_{k,t}\;\right)=-\frac{\partial {\tilde{L}}_{k,n}}{\partial T_{k,n}}\left(\;\mu_{k},T_{k,t}\;\right), \end{array}$$

is such that an algorithm sampling at each time *t* the cluster *X*_*k*_*t*__ with

(17) $$\begin{array}{c} k_{t}\in \underset{1\leq k\leq K}{\arg\;\max}\frac{T_{k,t}^{*}}{T_{k,t}}=\underset{1\leq k\leq K}{\arg\;\max\;}C_{k,t}^{i}\left(\;\mu_{k},T_{k,t}\;\right) \end{array}$$

would result in the optimal allocation of the budget *n*.

We have seen here an optimal allocation of the budget *n* that the optimist algorithm which will be defined in Section 4.3 could try to reach without the knowledge of the *μ*_*k*_ values. The criterion we derived only depends on the values of the parameters in each cluster and not the current labels given by the classifier. Considering them would lead to a better allocation since the allocation in a cluster could stop when the correct label is given.

#### 4.2.2. Data-Dependent Criterion

In this section, we show a criterion that leads to the optimal allocation of the budget *n* depending not only on the values of *μ*_*k*_ in each cluster, but also on the current labels given by the classifier.

We define a new global loss that is the current regret of the true risk:

(18) Lndμkk∈1,K,μ^k,nk∈1,K,Tk,nk∈1,K=∑k=1KwkLk,ndμk,μ^k,n,Tk,n,

with

(19) Lk,ndμk,μ^k,n,Tk,nRkμ^k,n−Rkμk=2μk−0.51μ^k,n≠μk.

The measure of performance is still the expected true risk but the value to be minimized is preferred to be run-dependent.

In order to minimize it, the selection strategy samples the cluster for which the expected decrease of the loss would be maximum. This criterion is thus the finite difference of the loss *𝔼*[Δ*L*_*k*,*t*_^*d*^] with

(20) ΔLk,td=Lk,tdμk,Tk,tμ^k,t+sTk,t+1,Tk,t+1−Lk,tdμk,μ^k,t,Tk,t,

where *s* is the label resulting from the sample and the expectation is taken on *s*.

However, this is a good strategy only if this criterion is strictly increasing with *T*_*k*,*n*_. We thus study the monotonicity of this criterion. We consider sampling *T* ^+^ more instances in cluster *k* with resulting average label ${\hat{\mu }}^{+}$. The new label given by the classifier will be $l_{k,t}^{+}=[\left(T_{k,t}{\hat{\mu }}_{k,t}+T^{+}{\hat{\mu }}^{+}\right)/\left(T_{k,t}+T^{+}\right)]$.

After *T* ^+^ samples, the expected decrease of the loss is

(21) EΔT+Lk,td=2μk−0.5Plk,t+≠μk−1μ^k,n≠μk.

Injecting the value of *l*_*k*,*t*_^+^,

(22) Plk,t+=1PTk,tμ^k,t+T+μ^+Tk,t+T+≥0.5=PT+μ^+≥Tk,t0.5−μ^k,t+T+2.

To shorten notations we use $D_{k,t}=2T_{k,t}\left(0.5-{\hat{\mu }}_{k,t}\right)$.

We know that ${\hat{\mu }}^{+}$ is drawn from a binomial distribution of parameter *μ*_*k*_ and *T* ^+^, thus

(23) Plk,t+=1=1−I1−μkT+−Dk,t+T+2,Dk,t+T+2+1,Plk,t+=0=I1−μkT+−Dk,t+T+2,Dk,t+T+2+1.

The criterion is not strictly increasing. In order to consider this constraint, we define another criterion which is a tight bound of the previous one. We first bound the following probabilities:

(24) Plk,t+=11−I1−μkT+−Dk,t2+1,T++Dk,t2=P1μk,T+,Tk,t,μ^k,t.

Equivalently,

(25) Plk,t+=0Plk,t+=0≤I1−μkT+−Dk,t2,T++Dk,t2+1=P0μk,T+,Tk,t,μ^k,t.

The criterion resulting from this bounds is strictly increasing but is not defined for all *T* ^+^. Indeed, in order to change the value of the label, the estimated mean has to move to the other side of 0.5. This often requires more than one sample (e.g., if we already sampled 10 instances and 8 were labeled 1, we need at least 6 new samples to have a chance to change the label given by the classifier). In order to get a bound defined for *T* ^+^ = 1 and strictly increasing with *T* ^+^, we make a linear interpolation between the value in *T* ^+^ = |*D*_*k*,*t*_| and the value in *T* ^+^ = 0 which is 0.

We thus define the actual criterion:

(26) ΔL~k,td=2wkμk−0.5Dk,t1μk=0P0μk,Dk,t,Tk,t,μ^k,t+1μk=1P0μk,Dk,t,Tk,t,μ^k,t−1μ^k,t≠μk.

The online allocation criterion is

(27) $$\begin{array}{c} C_{k,t}^{d}\left(\;\mu_{k},T_{k,t},{\hat{\mu }}_{k,t}\;\right)=-{\tilde{\Delta L}}_{k,t}^{d}, \end{array}$$

and it is such that an algorithm sampling at each time *t* the cluster *X*_*k*_*t*__ with

(28) $$\begin{array}{c} k_{t}\in \underset{1\leq k\leq K}{\arg\;\max\;}C_{k,t}^{d}\left(\;\mu_{k},T_{k,t},{\hat{\mu }}_{k,t}\;\right) \end{array}$$

would result in the optimal allocation of the budget *n*.

The criterion defined in this section leads to an optimal allocation of the budget *n* that the optimist algorithm which will be defined in the next section could try to reach without the knowledge of the *μ*_*k*_ values. It depends on the value of the parameters in each cluster as well as the current estimate of this parameter by the classifier.

### 4.3. Included Optimism

In this section we introduce two optimistic algorithms: OALC-DI (Optimistic Active Learning for Classification: Data Independent) which use the data-independent criterion and OALC-DD (Optimistic Active Learning for Classification: Data Dependent) which use the data-dependent criterion for optimal budget allocation defined in the previous sections. Both can be described by the same core algorithm. Neither criteria can be used as they are currently defined, for the active learning problem. Indeed, the value of *μ*_*k*_ in each cluster is not known in advance; otherwise, the correct label would be known as well. Also, it cannot directly replace those values by their estimation which could lead to clusters being turned down. This is a case of the exploration/exploitation tradeoff where the uncertainty about the true value of *μ*_*k*_ in each cluster has to be considered. Therefore, we design an optimistic algorithm that estimates those values and samples as close as possible to the optimal allocation.

Following the Optimism in the Face of Uncertainty approach, it builds a confidence interval on the criterion to be maximized and draw the arm for which the upper bound of this interval is highest. This is equivalent to saying it draws the arm for which the criterion is possibly the highest. As we know the shape of the distribution of the ${\hat{\mu }}_{k,t}$ values, the confidence interval is a Bayesian Credible Interval [21] which leads to tight bounds. The Bayesian Credible Interval is relative to a probability *δ* which allows for controling the amount of exploration of the algorithm. The core algorithm is presented in Algorithm 1. It takes one parameter *δ* and can be derived in two algorithms depending on the criterion used.

Let us show how to build the Bayesian Credible Interval. As each sample is drawn from a Bernoulli distribution, the estimated means follow a binomial distribution. Beta distributions provide a family of conjugate prior probability distributions for binomial distributions. The uniform distribution Beta(1,1) is taken as the prior probability distribution, because we have no information about the true distribution. Using the Bayesian inference,

(29) Pμk=x ∣ μ^k,t,Tk,t=xTk,tμ^k,t1−xTk,t1−μ^k,tBetaTk,tμ^k,t+1,Tk,t1−μ^k,t+1.

In the following *C*_*k*,*t*_ means either *C*_*k*,*t*_^*i*^ from (16) or *C*_*k*,*t*_^*d*^ from (27). Obviously,

(30) $$\begin{array}{c} P\left(\;C_{k,t}>e_{k}\;|\;{\hat{\mu }}_{k,t},T_{k,t}\;\right)=P\left(\;\mu_{k},C_{k,t}>e_{k}\;|\;{\hat{\mu }}_{k,t},T_{k,t}\;\right). \end{array}$$

Let *I*_*k*_ = {*μ*_*k*_∣*f*(*T*_*k*,*t*_, *μ*_*k*_) > *e*_*k*_}, then

(31) PCk,t>ek ∣ μ^k,t,Tk,t=∫x∈IkxTk,tμ^k,t1−xTk,t1−μ^k,tdxBetaTk,tμ^k,t+1,Tk,t1−μ^k,t+1.

The upper bound of the Bayesian Credible Interval is then

(32) $$\begin{array}{c} e_{k}\text{ s.t. }P\left(\;C_{k,t}>e_{k}\;|\;{\hat{\mu }}_{k,t},T_{k,t}\;\right)=\delta . \end{array}$$

In this section, we have shown two optimistic algorithms that share the same core. The difference lies in the full knowledge criterion used. One depends only on the value of the parameters of the distributions. The other one depends on both the value of the parameters and the current estimates of this parameter by the classifier. Both the resulting algorithms depend only on the estimates of the parameters.

The problem solved by those algorithm is the one that finds the best label to give to several separated clusters. This separation comes from the partition of a continuous instance space. A good hypothesis would be that the *μ*_*k*_ values do not vary fast and that neighbor clusters have close values of *μ*_*k*_. In order to speed up learning and to increase generalization, we could estimate *μ*_*k*_ considering neighbor clusters. This is the subject of next section.

## 5. Information Sharing Clusters

### 5.1. A Set of Partitions

The previous section introduces an active learning algorithm which is based on a partition of the instance space. Supposing this partition is given, it defines the best allocation of samples among its clusters that lead to the lowest true risk of the classifier also based on this partition. The best performance of the classifier still highly depends on the choice of the partition, which has no obvious solution. One way to improve the classifier's performance is to increase the number of clusters in the partition. But this slows learning as each cluster parameter has to be estimated independently. To counter that, we allow the classifier to generalize by letting neighbor clusters share information. In order to use the same approach as before, we consider the case of a committee of partitions. Each partition estimates the parameter of their clusters independently. Then, the local prediction of the label is determined by averaging the estimations of each partition.

Let **N** be a set of *m* partitions of the instance space:

(33) $$\begin{array}{c} N=\left\{\;N_{1},\ldots ,N_{m}\;\right\}, \end{array}$$

where ∀*j* ∈ {1,…, *m*}:

(34) $$\begin{array}{c} N_{j}=\left\{\;X_{1,j},\ldots ,X_{K_{j},j}\;\right\}, \end{array}$$

with the following properties:

(i) ∀*i* ∈ ⟦1, *K*_*j*_⟧ : *X*_*i*,*j*_ ≠ *∅*; no subset is empty,
(ii) ∪_*i*=1_^*K*_*j*_^ *X*_*i*,*j*_ = *X*; the subsets cover the whole instance space,
(iii) ∀(*i*, *k*) ∈ ⟦1, *K*_*j*_⟧^2^ : *i* ≠ *k*⇒*X*_*i*,*j*_∩*X*_*k*,*j*_ = *∅*; no subsets overlap.

Each partition may have a different number of subsets *K*_*j*_.

These partitions may come from random forests [22] or tile coding which is a function approximation method commonly used in the field of reinforcement learning [23]. The partitions must not change during the progress of the algorithm.

We write ${\hat{\mu }}_{i,j}=(1/W_{i,j})\sum_{t=1}^{n}1_{x_{t}\in X_{i,j}}y_{t}$, the average label in each cluster of each partition, and *W*_*i*,*j*_ = ∑_*t*=1_^*n*^1_*x*_*t*_∈*X*_*i*,*j*__, the number of samples in subset *j*.

Let us now define the thinnest partition *𝒩*, which is the partition resulting from overlapping all the partitions from **N**:

(35) $$\begin{array}{c} N=\left\{\;X_{1},\ldots ,X_{K}\;\right\} \end{array}$$

such that ∀*c* ∈ ⟦1, *𝒦*⟧, ∀(*x*_*a*_, *x*_*b*_) ∈ *𝒳*_*c*_^2^ : ∀*j* ∈ ⟦1, *m*⟧, ∃*i* ∈ ⟦1, *K*_*j*_⟧, *x*_*a*_ ∈ *X*_*i*,*j*_⇔*x*_*b*_ ∈ *X*_*i*,*j*_.

This means that two elements coming from the same subset of the thinnest partition necessarily come from the same subset in any partition of the set.

Each cluster *c* of this thinnest partition is associated with a Bernoulli distribution of parameter *μ*_*c*_.

We write ${\hat{\mu }}_{c}=(1/W_{c})\sum_{t=1}^{n}1_{x_{t}\in {𝒳}_{c}}y_{t}$, the average label in cluster *c*, and *W*_*c*_ = ∑_*t*=1_^*n*^1_*x*_*t*_∈*𝒳*_*c*__, the number of samples in cluster *c* of the thinnest partition. We write *W*_*c*,*i*,*j*_ = *W*_*c*_/∑_*c*=1_^*𝒦*^1_*𝒳*_*c*_⊂*X*_*i*,*j*__ *W*_*c*_, the relative importance of cluster *c* of the thinnest partition in cluster *i* of partition *j*. Note that ${\hat{\mu }}_{i,j}=\sum_{c=1}^{𝒦}1_{{𝒳}_{c}\subset X_{i,j}}W_{c,i,j}{\hat{\mu }}_{c}$.

**Figure 2:**
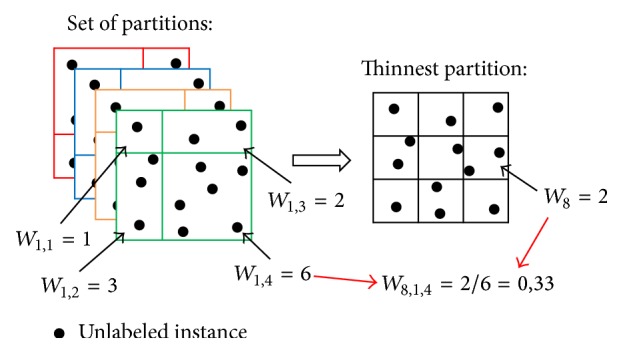
Representation of a set of partitions for a 2D instance space.

In order to understand what those values exactly are, an illustration is given on Figure 2. It shows an example of the partition of a two-dimensional instance space. The set is composed of four partitions of four clusters each. The resulting thinnest partition is, in this case, composed of nine clusters. The dots represent the unlabeled instances with which the weights of each clusters of each partition are computed. The influence of a cluster of the thinnest partition in the estimation of the mean value in one cluster of a specific partition of the set is also computed. What is not shown is how the influence of this last estimation on the final prediction is computed. It is 1/4 in this case because there are 4 partitions in the set.

The prediction of the label is cluster *c* that results from the averaging of estimations of each partition:

(36) $$\begin{array}{c} l_{c}=\left[\;\frac{1}{m}\overset{m}{\underset{j=1}{\sum }}\;\overset{K_{j}}{\underset{i=1}{\sum }}1_{X_{c}\subset X_{i,j}}{\hat{\mu }}_{i,j}\;\right]. \end{array}$$

This can also be written as

(37) $$\begin{array}{c} {\left(\;l_{1},\ldots ,l_{K}\;\right)}^{T}=\left[\;P\times {\left(\;{\hat{\mu }}_{1},\ldots ,{\hat{\mu }}_{K}\;\right)}^{T}\;\right], \end{array}$$

where the elements of *P* are ∀(*c*_1_, *c*_2_) ∈ ⟦1, *𝒦*⟧^2^

(38) $$\begin{array}{c} P_{c_{1},c_{2}}=\left[\;\frac{1}{m}\overset{m}{\underset{j=1}{\sum }}\;\overset{K_{j}}{\underset{i=1}{\sum }}1_{X_{c_{1}}\subset X_{i,j}}1_{X_{c_{2}}\subset X_{i,j}}W_{c_{2},i,j}\;\right]. \end{array}$$

Note that the size of the matrix *P* depends on the number of subsets in the thinnest partition. It may be very large if the initial partitions are not constrained. This is not a problem since a subset of the thinnest partition containing no instance from the training set causes its corresponding column of *P* to be null. It can therefore be removed. The size of *P* is thus limited by the number of instances in the training set.

In the active learning setting with a pool-based sampling scheme, the labels of the instances in the training set are not known in advance but are acquired sequentially. Although the pool of unlabeled instances is known initially, this allows us to compute the matrix *P* at the beginning of our algorithm and keep it until the end. This is different from the case where we consider only data already sampled, as done in Random Forests, and the matrix *P* has to be recomputed at each step.

Each cluster *𝒳*_*c*_ ∈ *𝒩* is an arm of a multiarmed bandit characterized by a Bernoulli distribution *ν*_*c*_ with mean value *μ*_*c*_. At each round, or time step, *t* ≥ 1, an allocation strategy selects an arm *c*_*t*_ ∈ ⟦1, *𝒦*⟧, which corresponds to picking an instance randomly in the cluster *𝒳*_*c*_*t*__ and receives a sample *y*_*t*_ ~ *ν*_*c*_, independently of the past samples. (*W*_*c*_)_*c*∈⟦1, *𝒦*⟧_ denote the weight of each cluster.

Then, if *T*_*c*,*t*_ = ∑_*s*=1_^*t*^1_*x*_*s*_∈*𝒳*_*c*__ is the number of samples taken in subset *c* up to time *t* and ${\hat{\mu }}_{c,t}=(1/T_{c,t})\sum_{s=1}^{t}1_{x_{s}\in {𝒳}_{c}}y_{s}$ if *T*_*c*,*t*_ ≠ 0 and 0.5, otherwise, is the mean of labels taken up to time *t*.

Let us define the classifier that gives to subset *c* the label

(39) $$\begin{array}{c} f\left(\;x\in X_{c}\;\right)=l_{c,t}=\left[\;\frac{\sum_{c^{\prime }=1}^{K}P_{c,c^{\prime }}{\hat{\mu }}_{c^{\prime },t}-0.5}{\sum_{c^{\prime }=1}^{K}P_{c,c^{\prime }}1_{T_{c}>0}}+0.5\;\right]. \end{array}$$

Note that it gives the same label as (37) where $l_{c,t}=\left[\sum_{c^{\prime }=1}^{𝒦}P_{c,c^{\prime }}{\hat{\mu }}_{c^{\prime },t}\right]$, and the only difference is that the value inside the [·] is reweighted such that it is 1 when all ${\hat{\mu }}_{c,t}$ are equal to 1.

### 5.2. Full Knowledge Criterion

In this section, we define the allocation of the budget in the full knowledge setting for the case of information sharing clusters. We also introduce a criterion that leads to this allocation. Again, those parameters are only used to define the allocation of the budget and not for the prediction of labels.

In this problem, the clusters are not independent. This means sampling an instance in a cluster affects the prediction of other clusters. The criterion has shown here the results of the myopic minimization of the true risk. With the weights of each clusters being estimated from the number of instances in the pool (labeled and unlabeled), the true risk is computed as the risk on the pool. To decide the next cluster to sample, we simulate sampling in each cluster and evaluate its expected impact on the risk. The selected cluster is thus the one which lowers the risk most.

Here, the risk is based on the binary loss:

(40) $$\begin{array}{c} L_{0/1}\left(\;y,f\left(\;x\;\right)\;\right)=\left\{\begin{array}{cc} 1, & \text{if }f\left(x\right)\neq y,\\ 0, & \text{otherwise.} \end{array}\right. \end{array}$$

Note that this loss is usually hard to use because of its nonconvex nature.

First, suppose that the label *l*_*c*,*t*_ is given to each subset by the classifier at time *t*. The true risk encountered in each subset is

(41) $$\begin{array}{c} R_{c}\left(\;l_{c,t}\;\right)=\left\{\begin{array}{cc} W_{c}\left(1-\mu_{c}\right), & \text{if }l_{c,t}=1,\\ W_{c}\mu_{c}, & \text{if }l_{c,t}=0. \end{array}\right. \end{array}$$

Note that the best risk is attained when *l*_*c*,*t*_ = [*μ*_*c*_].

Then, the true risk in each subset can also be written as follows:

(42) $$\begin{array}{c} R_{c}\left(\;l_{c,t}\;\right)=W_{c}\left|\;\mu_{c}-0.5\;\right|1_{l_{c,t}\neq \left[\mu_{c}\right]}+R_{c}\left(\;\left[\;\mu_{c}\;\right]\;\right). \end{array}$$

Let **T**_*t*_ = (*T*_1,*t*_,…, *T*_*𝒦*,*t*_)^*T*^ be the number of samples taken and ${\hat{\mu }}_{t}={\left({\hat{\mu }}_{1,t},\ldots ,{\hat{\mu }}_{𝒦,t}\right)}^{T}$ the current mean of labels, in each subset at time *t*. Let *P*_*c*_ be the *c*th row of *P*.

Let us remember that the labels given by the classifier come from (39)

(43) $$\begin{array}{c} R_{c}\left(\;T_{t},{\hat{\mu }}_{t},\mu_{c}\;\right)=W_{c}\left|\;\mu_{c}-0.5\;\right|1_{\left[P_{c}\times {\hat{\mu }}_{t}\right]\neq \left[\mu_{c}\right]}+R_{c}\left(\;\left[\;\mu_{c}\;\right]\;\right). \end{array}$$

Let us note

(44) Ttc=T1,t,…,Tc−1,t,Tc,t+1,Tc+1,t,…,TK,tT,μ^tc+=μ^1,t,…,μ^c−1,t,Tc,tμ^c,t+1Tc,t+1,μ^c+1,t,…,μ^K,tT,μ^tc−=μ^1,t,…,μ^c−1,t,Tc,tμ^c,tTc,t+1,μ^c+1,t,…,μ^K,tT.

Knowing that the probability for the next sample taken in subset *c* to be 1 is *μ*_*c*_, we have the following expected decrease in the risk incurred by sampling a new instance in subset *c*′ that we note

(45) Δc′RcTt,μ^t,μc=μc′RcTtc,μ^tc′+,μc−RcTt,μ^t,μc+1−μc′RcTtc,μ^tc′−,μc−RcTt,μ^t,μc.

With the global risk being

(46) $$\begin{array}{c} R\left(\;T_{t},{\hat{\mu }}_{t},\mu \;\right)=\overset{K}{\underset{c=1}{\sum }}R_{c}\left(\;T_{t},{\hat{\mu }}_{t},\mu_{c}\;\right), \end{array}$$

its expected decrease relatively to a new sample in subset *c* is

(47) $$\begin{array}{c} \Delta_{c^{\prime }}R\left(\;T_{t},{\hat{\mu }}_{t},\mu \;\right)=\overset{K}{\underset{c=1}{\sum }}\Delta_{c^{\prime }}R_{c}\left(\;T_{t},{\hat{\mu }}_{t},\mu_{c}\;\right). \end{array}$$

We then make a myopic minimization of the risk. Thus, at time *t*, our full knowledge algorithm samples the subset

(48) $$\begin{array}{c} c_{t}=\underset{c}{\arg\;\min\;}C_{c}\left(\;T_{t},{\hat{\mu }}_{t},\mu \;\right), \end{array}$$

where

(49) $$\begin{array}{c} C_{c}\left(\;T_{t},{\hat{\mu }}_{t},\mu \;\right)=\Delta_{c}R\left(\;T_{t},{\hat{\mu }}_{t},\mu \;\right) \end{array}$$

is the criterion for the online allocation of the budget *n*.

Through this section, we have seen an online criterion, based on the maximum expected decrease in terms of risk, which a full knowledge algorithm would use to sample. The hypothesis made about the knowledge of the *μ*_*c*_ values is unrealistic because if they were known, the classification would be obvious; however, it allowed us to determine a good allocation strategy that our partial knowledge algorithm tries to attain. In the next section, we remove this hypothesis and use an optimistic approach to estimate the *μ*_*c*_ and at the same time allocate samples as close as possible to the full knowledge allocation.

### 5.3. Included Optimism

In this section, we introduce two optimistic algorithms based on the full knowledge criterion of the previous section. The values of *μ*_*c*_ are not given, so the selection strategy cannot use them directly. Although the labels acquired during the sampling process allow us to estimate them. The simple replacement of the true values with their estimates is unjustified and could lead to a bad allocation.

Instead, we should consider the exploration/exploitation tradeoff. More than the only estimates, we are able to compute a distribution on the belief of *μ*_*c*_. A Bayesian Credible Interval, relatively to a probability *δ*, can thus be computed on the values of *μ*_*c*_, as well as on the criterion.

Referring to the Optimism in the Face of Uncertainty approach, we define a selection strategy that samples at every time step the cluster for which the upper bound of the Bayesian Credible Interval is highest. This leads to an allocation of the samples as close as possible to the Full Knowledge allocation even though the true value of *μ*_*c*_ is not known.

Our first algorithm is OEMAL (Optimistic Error Minimization for Active Learning). It uses the set of partition as defined in the previous section. The classifier is only defined by the matrix *P* representing the influence of clusters on each other. The number of clusters in the thinnest partition is not limited. One particular case of OEMAL is when its clusters contain at most one instance from the pool. This makes *P* act as the covariance matrix used in kernel methods.

Our second algorithm is OEMAL-k (Optimistic Error Minimization for Active Learning: kernel version). It takes as input any covariance matrix and use it as the matrix *P* in OEMAL. This broadens the scope of the classifiers that can be used. Note that, in order to use Optimism in the Face of Uncertainty in a proper way, the matrix *P* still must not change. The rest of the section works for both algorithms.

Our algorithm is displayed in Algorithm 2. It takes one parameter *δ* which allows us to control the level of exploration used by our algorithm.

At any time, we are able to compute a Bayesian Credible Interval on the value of the parameters of each cluster. Each subset *c* of the thinnest partition is associated with a Bernoulli distribution of parameter *μ*_*c*_. Thus, the estimated means are drawn from Binomial distributions. Beta distributions provide a family of conjugate prior probability distributions for Binomial distributions. With the prior probability distribution being Beta(1,1) (uniform distribution), by Bayesian inference,

(50) $$\begin{array}{c} P\left(\;\mu_{c}\;|\;{\hat{\mu }}_{c}\;\right)=\text{Beta}\left(\;T_{c}{\hat{\mu }}_{c}+1,T_{c}\left(\;1-{\hat{\mu }}_{c}\;\right)+1\;\right). \end{array}$$

However, this inference does not consider observations from the neighbor clusters. Let us define **m** = (*m*_1_,…, *m*_*𝒦*_)^*T*^ with ∀*c* ∈ ⟦1, *𝒦*⟧,

(51) $$\begin{array}{c} m_{c}=\frac{\sum_{c^{\prime }=1}^{K}P_{c,c^{\prime }}\mu_{c^{\prime }}-0.5}{\sum_{c^{\prime }=1}^{K}P_{c,c^{\prime }}1_{T_{c}>0}}+0.5, \end{array}$$

which is the decision criterion of the classifier defined in (39). In order to get an early guess about the value of ***μ***, we chose to infer **m** instead, which aims to approach ***μ***, and use it in place of ***μ***. Even though **m** ≠ ***μ*** and we may lose some accuracy, this is necessary for our algorithm to work well.

First, let us state that $\hat{m}$ results from a sum of independent Binomial distributions; therefore assuming that the number of nonnull elements in each row of *P* is large enough, we can approximate that $\hat{m}$ follows a normal distribution.

The normal distribution provides a family of conjugate prior probability distributions for the normal distributions; thus, our belief about **m** follows a normal distribution with a mean of ***μ*** ^*N*^ = (*μ*_1_^*N*^,…, *μ*_*𝒦*_^*N*^)^*T*^ with ∀*c* ∈ {1,…, *𝒦*},

(52) $$\begin{array}{c} \mu_{c}^{N}=\frac{\sum_{c^{\prime }=1}^{K}P_{c,c^{\prime }}{\hat{\mu }}_{c^{\prime },t}-0.5}{\sum_{c^{\prime }=1}^{K}P_{c,c^{\prime }}1_{T_{c}>0}}+0.5 \end{array}$$

and a standard deviation **s** ^*N*^ = (*s*_1_^*N*^,…, *s*_*𝒦*_^*N*^)^*T*^ with ∀*c* ∈ {1,…, *𝒦*},

(53) $$\begin{array}{c} s_{c}^{N}=\sqrt{\overset{K}{\underset{c^{\prime }=1}{\sum }}{\left(\frac{P_{c,c^{\prime }}}{\sum_{c^{\prime }=1}^{K}P_{c,c^{\prime }}1_{T_{c}>0}}\right)}^{2}v_{c^{\prime }}^{B}} \end{array}$$

with $v_{c^{\prime }}^{B}=(T_{c^{\prime }}^{2}{\hat{\mu }}_{c^{\prime }}(1-{\hat{\mu }}_{c^{\prime }})+T_{c^{\prime }}+1)/{\left(T_{c^{\prime }}+2\right)}^{2}\left(T_{c^{\prime }}+3\right)$, the variance of the Beta distribution.

Then,

(54) $$\begin{array}{c} P\left(\;m\;|\;\hat{\mu }\;\right)=N\left(\;\mu^{N}\left(\;\hat{\mu },T\;\right),s^{N}\left(\;\hat{\mu },T\;\right)\;\right). \end{array}$$

This algorithm, simulates the sampling of a new instance in order to estimate the resulting gain in risk. Within each simulation the distribution of the belief is updated by taking into account the new sample. Then, this new distribution of the belief is used to compute a distribution on the gain in risk.

Let us note *y*_*s*_ is the label of the instance sampled in the simulation and

(55) $$\begin{array}{c} {\hat{\mu }}_{t}^{c}={\left(\;{\hat{\mu }}_{1,t},\ldots ,{\hat{\mu }}_{c-1,t},\frac{T_{c,t}{\hat{\mu }}_{c,t}+y_{s}}{T_{c,t}+1},{\hat{\mu }}_{c+1,t},\ldots ,{\hat{\mu }}_{K,t}\;\right)}^{T}. \end{array}$$

We thus replace

(56) $$\begin{array}{c} P\left(\;C_{c}\left(\;T_{t},{\hat{\mu }}_{t},\mu \;\right)=Q\;|\;{\hat{\mu }}_{t}\;\right) \end{array}$$

by

(57) Pys ∣ μc=mcPmc ∣ μ^t·PCcTt,μ^t,m=Q ∣ μ^tc=∑∑c′qc′=QPys ∣ mcPmc ∣ μ^t·∏c′PΔcRc′mc′=qc′ ∣ μ^tc.

In order to compute this value we define two methods.

**Method 1 (only for OEMAL).**

(i) Draw a high number of instantiations of the belief from $\mathbb{P}(m|\hat{\mu })$.

(ii) Compute for each case the resulting value of the criterion.

(iii) Estimate the distribution of the criterion.

In our experiments, the set of partitions **N** we used is such that every cluster of the thinnest partition contained at most one instance from the pool. Thus, the value of *μ*_*c*_ is contained in {0,1}.

**Method 2 (if ∀*c* ∈ ⟦1, *𝒦*⟧*W*_*c*_ = 1).**

Compute the probability of *μ*_*c*_ being 0 as follows:

(58) $$\begin{array}{c} P\left(\;\mu_{c}=0\;|\;{\hat{\mu }}_{t}\;\right)=\Phi \left(\;0.5,\mu^{N}\left(\;{\hat{\mu }}_{t},T_{t}\;\right),s^{N}\left(\;{\hat{\mu }}_{t},T_{t}\;\right)\;\right), \end{array}$$

with Φ being the cumulative distribution function of the normal distribution:

(59) ∑∑c′qc′=QPys ∣ μcPμc ∣ μ^t·∏c′PΔcRc′μc′=qc′ ∣ μ^tc

with

(60) $$\begin{array}{c} \Delta_{c}R_{c^{\prime }}=1_{\mu_{c^{\prime }}\neq l_{c^{\prime },t}^{c}}-1_{\mu_{c^{\prime }}\neq l_{c^{\prime },t}} \end{array}$$

and $l_{c^{\prime },t}^{c}\left({\hat{\mu }}_{t}^{c}\right)$ and $l_{c^{\prime },t}\left({\hat{\mu }}_{t}\right)$ from (39).

The simulation of a sample can lead to two states of the classifier. Depending on this state, the true risk is the sum of the true risk in each cluster, with at most two cases each (*q*_*c*′_ ∈ {−1,1} or *q*_*c*′_ = 0), depending on the value of *μ*_*c*_ in each cluster. We can then compute the probability of a value of the criterion by combining the probability of each case. As the difference of the true risk for one cluster is 0 whenever the prediction of the classifier does not change, we only consider clusters for which the prediction of the classifier changes. With this, the computation of the distribution of the criterion can be done in a reasonable time.

Having computed the distribution of the criterion, we can compute the upper bound of the Bayesian Credible Interval, which is

(61) $$\begin{array}{c} e_{c}=\underset{\epsilon }{\arg\;\min\;}P\left(\;C_{c,t}>\epsilon \;|\;{\hat{\mu }}_{t},T_{t}\;\right)\leq \delta . \end{array}$$

In this section, we derived two optimistic algorithms that address the active learning problem for classification. OEMAL considers a set of partitions of the instance space as well as the thinnest partition, resulting from overlapping this set. OEMAL-k considers a kernel. They both find the cluster of the thinnest partition for which sampling in it results in the greatest decrease of the true risk.

### 5.4. Computational Complexity

Let *𝒦* be the number of clusters of the thinnest partition, and let *𝒦*_*U*_ be the number of clusters with at least one unlabeled instance. The computation of the criterion requires *𝒪*(*𝒦* ^2^) time complexity.

Using Method 1, let *n*_its_ be the number of instantiations of the belief. The selection of the next cluster thus requires *𝒪*(*𝒦* ^2^ *𝒦*_*U*_ *n*_its_) time complexity. Indeed, at each time step, it computes the criterion for *n*_its_ values of the parameters drawn from the posterior and for the simulation of sample in each one of *𝒦*_*U*_ clusters.

In the case where each cluster contains only one instance from the pool, the decrease in risk in one cluster can be 0 if the predicted label does not change, or 1 or −1 depending on the true label, if it changes. Let *n*_changes_ be the number of clusters seeing its label change for the considered simulation of sample.

Using Method 2, the computational complexity of the combinatorial procedure is *𝒪*(*n*_changes_^2^). Thus, the selection of the next cluster requires a computational complexity of *𝒪*(*𝒦* ^2^ *𝒦*_*U*_ *n*_changes_^2^).

Added to the fact that Method 2 is more precise than Method 1 because the computed cumulative distribution is exact, it is also faster, because *n*_changes_ rapidly decreases while acquiring more samples.

Note that the number of partitions in the set is not involved in the computational complexity. Indeed, they are only involved in the computation of the relations between subsets of the thinnest partition which is made beforehand. This is another advantage compared with the use of random forests, where each tree has to be recomputed at every time step.

## 6. Evaluation

In this section, we evaluate the algorithms introduced in the previous sections. Each of the evaluation will be the scope of a comparison between our algorithms and state-of-the art algorithms. Algorithms are evaluated on two different benchmarks depending on the classifier used.

In the case of independent clusters, either the instance space is already discrete, or it is continuous and must be partitioned. In the latter, the partition has to be chosen before taking any samples and cannot be changed after that. The predicted label in each cluster depends only on samples drawn in it. Thus, the number of clusters is limited by the budget of samples and by a desired good learning rate. This makes the partition rough and the classifier noncompetitive on real world datasets. However, the small number of parameters needed to represent any real world problem allows us to build a representative benchmark.

In the case of information sharing clusters, a partition is also given beforehand but the predicted label in each cluster may depend on all the samples. The number of clusters is thus not limited as before, and an extremely refined partition, where each cluster contains only one instance from the pool, may be used. Thus, this algorithm may be evaluated on real world problems with a continuous instance space.

### 6.1. Independent Clusters

#### 6.1.1. Practical Implementation and Experimental Setup

Two optimistic algorithms were introduced in the context of independent clusters. Each one based on different full knowledge criteria: the first one defines a one-shot allocation of the samples, which means that the optimal number of samples to take in each cluster is only function of its parameter and of the budget, and the second one defines a fully sequential allocation of the samples, which means that the optimal number of points to take in each cluster depends also on the samples drawn.

The performance of the algorithms closely depends on the choice of the partition. Clusters may regroup instances with a great dispersion of labels, either because the instances are subject to a lot of noise, or because the mean label varies rapidly. In this second case, a better discretization of the instance space causes a better true risk when the correct label is given. But this implies increasing the number of clusters, and for the prediction of the label to be equivalently accurate, we need a higher budget. As the problem of the choice of the partition is not studied here, the use of our algorithm on real world datasets with a continuous instance space would not be competitive with methods designed for this problem. Instead, we show that, given any partition, the algorithm performs better than other algorithms with the same constraints.

In this problem, where the classifier predicts one label per cluster, the location in the cluster of the sampled instance is of no interest. Therefore, every cluster can be seen as a pool of instances returning labels with a certain proportion. With the labels being either 0 or 1, this leads to a representation of clusters as Bernoulli distribution only characterized by one parameter. The relative position of the clusters is also of no interest for the classifier. Consequently, every problem our algorithm could encounter is only characterized by

(i) the number of clusters *K*,
(ii) the parameter of the Bernoulli distribution associated with each cluster (*μ*_*k*_)_*k*∈⟦1, *K*⟧_,
(iii) the weight of each cluster (*w*_*k*_)_*k*∈⟦1, *K*⟧_.

If two problems share the same characteristics, our algorithms will act the same on them.

Our first benchmark is thus to generate randomly a set of problems by drawing random parameters from the above definition. Then, the tested algorithms are launched on each problem of the set, and their true risk is recorded at every time step. The current predicted label for each cluster is compared to the correct one (the round value of *μ*_*k*_), and the true risk is the weight sum of this logical comparison. Note that because the problems are built in, the true parameter is known and we do not need a test set. Likewise, the samples are directly drawn from the distributions and not drawn from a pool. The global performance of the algorithms results from averaging the true risk at each time step. In our experiments, the benchmark contains 1000 problems generated with

(i) *K* drawn uniformly in ⟦1,50⟧,
(ii) (*μ*_*k*_)_*k*∈⟦1, *K*⟧_ drawn uniformly in [0,1]^*K*^,
(iii) (*w*_*k*_)_*k*∈⟦1, *K*⟧_ drawn uniformly in [0,1]^*K*^.

The weights are normalized, but this is just anecdotal as it does not affect the behavior of the algorithms. We use a budget of 1000 samples. The results are displayed with the time step range starting at 100 in order to be able to differentiate algorithms.

Our second benchmark comes from the fact that not all state-of-the-art algorithms consider the weight given to clusters. In order to make sure that the good performance of our algorithms is not only due to this consideration, in this benchmark the weights are equal for all clusters. The problem of allocation consists in defining which cluster has priority against another. Thus, any problem is a subproblem of the one containing an infinite number of clusters containing all the values possible for the parameter. Then, we generate one problem containing many clusters with the widest variety of parameters. In our experiments, this problem is generated with

(i) *K* = 100,
(ii) ∀*k* ∈ ⟦1, *K*⟧, *μ*_*k*_ = *k*/*K*,
(iii) ∀*k* ∈ ⟦1, *K*⟧, *w*_*k*_ = 1/*K*.

We run the algorithms 1000 times on this problem to face the randomness of the samples and average their true risk at every time step. The results are displayed with the time step range starting at 100.

To demonstrate the effectiveness of our algorithms, we compare them with existing state-of-the-art methods.

*Random Sampling*. This is the simplest baseline; at every time step the sampled cluster is drawn uniformly in ⟦1, *K*⟧. Any active learning algorithm should do better than that.

*Uniform Sampling*. This is another simple baseline, and the clusters are sampled uniformly. At every time step the sampled cluster is drawn uniformly among the least sampled ones.

*Monte Carlo Upper Confidence Bound (MC-UCB) (see [20])*. This algorithm also uses an optimistic approach, although it is not originally designed for classification. It aims to estimate uniformly well the parameter of the distribution in each cluster. We use this estimation to predict the label. This algorithm also considers a weight for each cluster.

*EffECXtive (see [12])*. This algorithm tries to identify the best hypothesis from a set by successively sampling the instance for which the expected reduction in weight is the greatest. The weight is defined as the sum of the squared probabilities of hypotheses. In our case, a hypothesis is characterized by the label given to each cluster, and the probability of a hypothesis is

(62) $$\begin{array}{c} P\left(\;h={\left(\;y_{k}\;\right)}_{k\in ⟦1,K⟧}\;\right)=\overset{K}{\underset{k=1}{\prod }}P\left(\;\left[\;\mu_{k}\;\right]=y_{k}\;|\;{\hat{\mu }}_{k}\;\right), \end{array}$$

where $\mathbb{P}\left(\left[\mu_{k}\right]=1|{\hat{\mu }}_{k}\right)={ℐ}_{0.5}(T_{k}{\hat{\mu }}_{k}+1,T_{k}(1-{\hat{\mu }}_{k})+1)$ with *ℐ*_*x*_(*a*, *b*) being the regularized incomplete beta function.

The three optimistic algorithms evaluated in this section, namely, MC-UCB, OALC-DI, and OALC-DD, use a parameter *δ*. This parameter has been tuned by using a grid search.

#### 6.1.2. Evaluation

**Figure 3:**
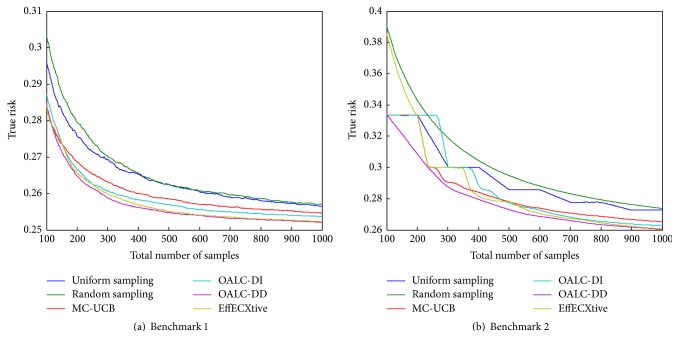
Evaluation of algorithms in the case of independent clusters.

First, we evaluate the performance of the algorithms on Benchmark 1. The results of the evaluation are displayed on Figure 3(a).

We first compare MC-UCB with OALC-DI. MC-UCB is an optimistic algorithm which tries to estimate uniformly well the mean value of the distribution in each cluster. This can be related to active learning in the case of regression. A good estimate of the mean value leads to a good prediction for the label. Therefore, this algorithm may be used on a classification problem even though it may not be the best. Indeed, it will spend effort to be precise on the estimation of the mean value even if this precision is of no use for the decision of the label. Those efforts could have been spent in a different cluster where the label uncertainty is larger. OALC-DI is the closest of our algorithms to MC-UCB as they both consider a Full Knowledge criterion that gives the optimal allocation of the budget without knowing the results of the samples. The two differ in that OALC-DI is specifically designed for classification. We can see that OALC-DI shows a significant improvement over MC-UCB, which indicates that working with an algorithm adapted for classification is not to be neglected.

We then compare OALC-DI and OALC-DD. Those two algorithms aim both to minimize the same objective function, which is the expected true risk of the classifier. Note that using directly the true risk based on the binary loss is usually avoided by minimizing a convex proxy of it. Although the second one is based on a full knowledge criterion that takes the current state of the classifier, depending on the results of the samples so far, into account whereas the first one is not. We can see that, as expected, the version of our algorithm based on a data-dependent full knowledge criterion behaves better than the one based on a data-independent one. Note that even though the difference in performance is only of 0.0016 at time step 1000, which means that 0.16% of the instances will be classified better, the number of time steps required to attain the performance of OALC-DI at time step 1000 is of 625 for OALC-DD, which is a save of 37.5% of the labeled instances.

Finally, let us take a look at the performances of OALC-DD and EffECXtive. Both algorithms are very similar as they greedily minimize an expected objective function. The difference is that EffECXtive uses a proxy of the true risk, the Rényi entropy, whereas OALC-DD, by allowing it to depend on the true parameters of the distribution before being optimist regarding it, is closer to the actual true risk. Note that EffECXtive, in our adaptation to a partition of the instance space, does not take into account relative importance given to clusters (weights), which are not trivial to include. The results show that OALC-DD performs slightly better than EffECXtive.

Let us now evaluate the performance on Benchmark 2. The results are displayed in Figure 3(b). This problem contains more clusters than any other problem in Benchmark 1. A higher budget is thus needed to attain comparable performances. Still, the range of time steps does not change. This allows us to focus on the first phase of the algorithms. In this problem, the weights are equal for all clusters, so that the quality of an algorithm is not only based on the fact it has this feature. In this problem, the best true risk is equal to 0.25.

Note that this range starts at 100 labeled examples. But since most algorithms need every cluster to be sampled at least once, at time step 100, each one of the 100 clusters will be sampled once, and this is for every algorithm, apart from random sampling. This is why every algorithm performance starts at the same level. We thus do not lose much by starting at this time step. The results of the first sample are 0 or 1 leading to the same precision on the prediction, and some algorithms need every cluster to be sampled twice, and this is why the performance at time step 200 is the same for most algorithms. EffECXtive performance at 100 time step is not the same as others as it samples indifferently clusters with no samples and clusters with one sample. But it has the same performance as others at time step 200. OALC-DD prefers to sample clusters that have received two samples 0 and 1 than one sample, which appears as a good behavior relatively to the results.

We can see that OALC-DD has better performance than all other algorithms at every time step.

### 6.2. Information Sharing Clusters

#### 6.2.1. Practical Implementation and Experimental Setup

Two other optimistic algorithms were introduced. Instead of one partition, OEMAL uses a set of partitions which all compute the estimates independently and merge to predict the final label. The thinnest partition was defined, which is the partition resulting from the intersection of all the partitions in the set. We have seen that we could use a representation of the classifier which involves only the clusters of the thinnest partition and a matrix *P* which tells how much the estimate in one cluster plays a role in predicting the label in another cluster. Using a set of partitions is thus equivalent to one partition with clusters that share information.

The classifier used by our algorithm shares some resemblance with Random Forests [22] as it uses a set of partitions and average of the prediction criterion of each one of them. In our algorithm, the set of partitions has to remain the same throughout the progress. Whereas in Random Forests, they are recomputed at each step. Hence, it cannot adapt to the received labels and must be defined at the beginning. We use purely random partitions of the instance space that are not based on the instances in the pool. It is thus more closely related to tile coding which is a function approximation method commonly used in the field of Reinforcement Learning [23].

The nature of the partitions in the set and its number was not defined in the algorithm. In fact, the algorithm works given any set of partitions. The performances of the classifier clearly depend on the partitions in the set; for example, if all the partitions in the set are the same, then the problem is reduced to the one partition problem. But, as long as partitions are diverse, their shape is not as determinant as before. In the context of one partition, the number of clusters in it could not be too large because the belief on the parameter which guided the selection strategy was only based on observations in its cluster. Now, the number of clusters in the thinnest partition has no limitation. This allows us to work with a continuous instance space without the loss in performance incurred by the choice of the partition.

The partitions we use consist of 7 successive random splits of the instance space along random dimensions. The number of partitions in the set is 10.000. At the end, the clusters of the thinnest partition contain either 1 or no instance from the pool.

OEMAL-k replaces the matrix *P* in OEMAL by a covariance matrix relatively to a kernel. In the experiments, we use a Gaussian kernel covariance matrix:

(63) $$\begin{array}{c} P_{i,j}=e^{-{\left|x_{i}-x_{j}\right|}^{2}/2s^{2}}. \end{array}$$

The scale parameter *s* has been tuned to give the best performance with a full training set.

We thus evaluate our algorithm on several real world datasets from the UCI Machine Learning Repository [24]. The four datasets used in this paper are Australian, Diabetes, Heart, and Wdbc. In all those datasets, the instances belong to a continuous instance space. In each run of experiments, the dataset is randomly divided into two. The first half is used as the pool of unlabeled instances in which the algorithm is allowed to pick, while the second half is used as the test set. At each time step, the true risk of the current prediction is estimated via the test set and recorded. The global performance of an algorithm at each time step is computed as the average of the performance among the runs. In our experiments, the number of runs is set to 1.000. The parameter *δ* is tuned for every dataset using a grid search.

We compare our algorithm with existing state-of-the-art method and some baselines.

*Random Sampling*. This is the simplest baseline. At each time step a random instance is drawn from the pool of unlabelled instances.

*Full Knowledge*. This is the best algorithm we can do. At each time step, an instance is selected according to the full knowledge criterion. The values of the parameters are used; thus, it is unrealistic but it serves to show how well the exploration/exploitation tradeoff is achieved. 

*Uncertainty Sampling (see [7])*. This is the most common active learning algorithm. At each time step, the instance which is most uncertain about how to label is selected.

#### 6.2.2. Evaluation

**Figure 4:**
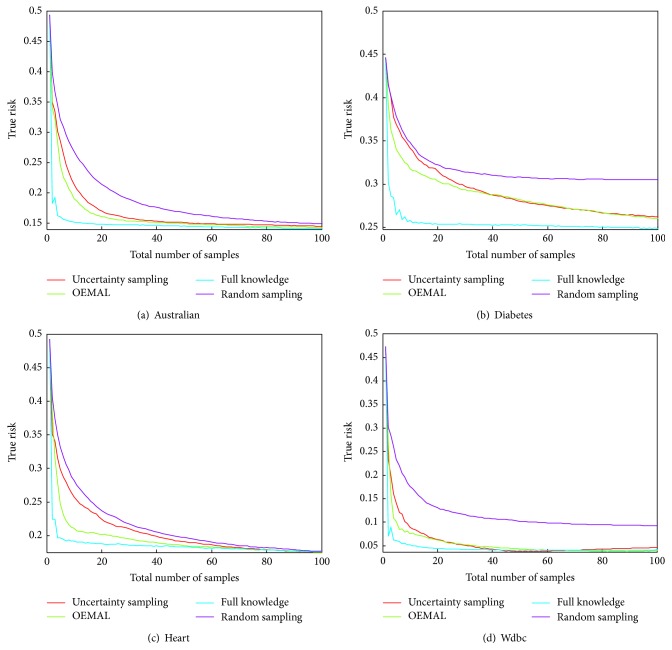
Evaluation of algorithms in the case of information sharing clusters.

Figure 4 displays the results of the evaluation for the four following datasets: Figure 4(a) Australian, Figure 4(b) Diabetes, Figure 4(c) Heart, and Figure 4(d) Wdbc.

We built our optimistic algorithm by first defining a full knowledge criterion which guides the allocation of samples in the case where the true values of the parameters are known from the beginning. This is then used by the algorithm as a target allocation to attain in the case where those values are unknown. The performances of OEMAL are thus limited by those of the full knowledge allocation. We display the performances of the Full Knowledge allocation as a baseline for the Optimistic algorithm. If they both have the same performances, then the only way to improve the algorithm is to design a better Full Knowledge criterion. Otherwise, the way to manage uncertainty is to improve. In other words, it tells if the exploration/exploitation tradeoff is well achieved. The full knowledge allocation, for its part, sees its performances limited by the classifier, which may not have better performances given any set of instances of size given by the time step. Also, whereas in the dependent clusters case the full knowledge allocation was known to be optimal, meaning that we could not achieve better performances with a different allocation, this is not the case anymore. Indeed, the myopic minimization of the risk has no guaranty to lead to the optimal allocation. For example, the best performances of the classifier at time step 2 could be achieved by the inclusion of a pair of instances that does not contain the instance that leads to the best performance at time step 1. Although the empirical results show that using a myopic minimization in full knowledge performs quite well, we can see that on the Wdbc dataset Figure 4(d), for a short period around 55 labeled instances, uncertainty sampling achieves slightly better performance than the full knowledge allocation. The optimistic algorithm can not thus do better on this period. Still, we can see that it outperforms it on the 20 first samples as well as it keeps high performance on the end while Uncertainty Sampling seems to lose accuracy.

**Figure 5:**
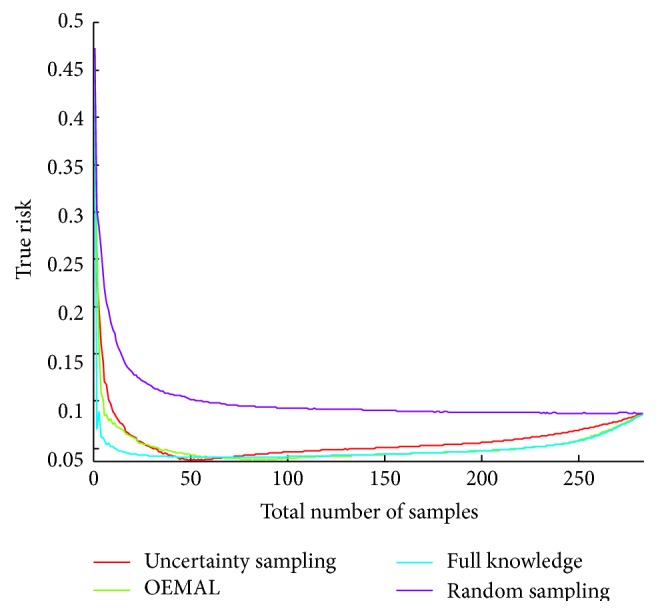
Evaluation of the algorithms on Wdbc until all the instances of the pool are sampled.

This last phenomenon can be explained. Let us look at Figure 5 where we display the results for the Wdbc dataset with the range of time steps extended to show the behavior of the algorithms until the last sample of the pool is retrieved. We can see the performance of all the algorithm decreases while approaching the end. We may think that the performance can only increase with the number of samples taken into account by the classifier. We know that this is not necessarily true, particularly if the classifier overfits. Also, in active learning, one can select a subset of instances that achieves better performance than when taking all the instances from the pool. The question of a stopping criterion has already been studied in order to avoid spending useless resources. Here, we see that it is even more crucial because it could lead to better performances. In OEMAL, the criterion used represents the maximum decrease of the true risk one can expect by taking a particular sample. Thus, if the value of the criterion is less than 0, this means that there is a high probability (1 − *δ*) that the true risk will not decrease but increase. In this case, it is preferable not to sample this instance. But if the maximum criterion is less than 0, it is preferable not to sample at all. We use this as a stopping criterion. We compare the final performances of our algorithm with and without the stopping criterion for different datasets in Table 1.

Two of the four datasets see their true risk increase at the end, Diabetes and Wdbc. We can see that the use of a stopping criterion is efficient in those cases and does not greatly alter the performances in other cases.

**Figure 6:**
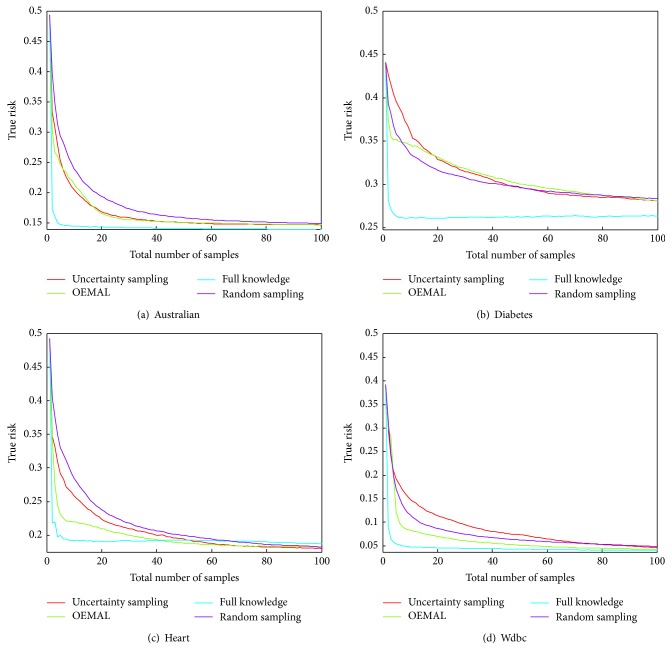
Evaluation of algorithms in the case of information sharing clusters.

The matrix *P* appearing in the classifier we used so far is derived from the use of a set of partitions. The value in each cell of the matrix corresponds to the weight of each estimation in each prediction. We saw that it was possible to use clusters of the thinnest partition that contain only one instance from the pool. Instances are linked to others through *P*. Inherently, in this case *P* fully specifies the data manifold structure. The theory that leads to OEMAL is built upon this set of partition, but we can imagine using a matrix *P* of any kind, specifying other weights between instances. OEMAL may still work in this context. Particularly, we can adapt this algorithm to the active learning of kernel classifiers. We thus use a Gaussian kernel covariance matrix for *P*. We now denote the kernel version of OEMAL by OEMAL-k. The results are displayed in Figure 6.

We can see that OEMAL-k always does better than uncertainty sampling. Even in the Wdbc dataset where uncertainty sampling performs worse than random sampling, OEMAL-k manages to get better performance. One important thing in active learning is the choice of the classifier. With a well-fitted classifier, even the random sampling strategy could perform better than the best active learning strategy on a poor classifier. It is thus convenient that our algorithm is not limited only to one kind of classifier and can easily generalize to kernel classifiers. For example, on the Wdbc dataset Figures 4(d) and 6(d), the random sampling strategy performs significantly better when using a kernel than a set of partitions, and OEMAL-k keeps improving the results. On the other hand, on the Diabetes dataset Figure 6(b), the random sampling strategy is also better with the kernel classifier, but OEMAL performs better than OEMAL-k. As we have seen in Figure 5, the active learning strategy of the best performance for the classifier may be achieved with only a subset of instances. Maybe the former classifier had a better potential than the last. This is confirmed by the performance of the full knowledge criterion.

In this section, we evaluated the performance of OEMAL which is built on this approach on several real world datasets. Thus, we demonstrated that the* Optimism in the Face of Uncertainty* approach can be used for active learning in classification. We saw that it performed comparatively well to a famous state-of-the art algorithm. We also evaluated OEMAL-k and showed that our algorithm could be generalized to kernel methods or any graph based method where the instances are linked to otherr by a weight matrix *P*.

## 7. Conclusion

In this paper, we show that the problem of active learning in classification can be studied through the eye of Optimism in the Face of Uncertainty. This has the advantage to allow the selection criterion to be defined as close as possible to the evaluation function. It introduces three error minimization algorithms which use this approach. The experiments, conducted on built-in problems as well as real world datasets, show that they perform comparatively well to state-of-the-art methods. An extension of the last algorithm shows that it can be generalized to other kernels. We however constrained the matrix *P* to remain the same all along the progress of the algorithm. Our perspective is to work with a changing matrix *P* such as in kernel regression or Gaussian processes where the variance of the estimates is given.

## Figures and Tables

**Algorithm 1:**
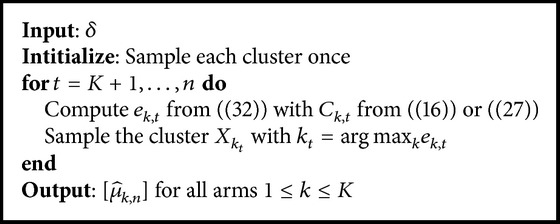
Core algorithm.

**Algorithm 2:**
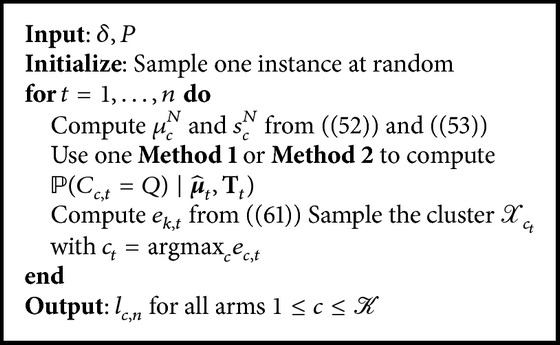
OEMAL/OEMAL-k.

**Table 1:** Final true risk of the classifier using OEMAL with or without using a stopping criterion.

Dataset	OEMAL with stopping criterion	OEMAL without stopping criterion
Australian	0.1367	0.1382
Diabetes	0.2422	0.3095
Heart	0.1724	0.1722
Wdbc	0.0397	0.0860
